# Values and practice of collaboration in a mental health care system in the Netherlands: a qualitative study

**DOI:** 10.1186/s13033-023-00584-9

**Published:** 2023-06-08

**Authors:** Suzanne J.C. Kroon, Manna A. Alma, Meike Bak, Lian van der Krieke, Richard Bruggeman

**Affiliations:** 1 Department of Psychiatry, Rob Giel Research Center, University of Groningen, University Medical Center Groningen, Groningen, The Netherlands; 2 Department of Health Sciences, Applied Health Research , University of Groningen, University Medical Center Groningen, Groningen, The Netherlands

**Keywords:** Collaboration, Ecosystem, Mental health care system, Qualitative study, Transition

## Abstract

**Background:**

To offer optimal care, the mental health system needs new routes for collaboration, involving both interprofessional and interorganizational aspects. The transition from intramural to extramural mental health care has given rise to new dynamics between public and mental health care, introducing a challenge for interprofessional and interorganizational collaboration. This study aims to determine values and expectations of collaboration and to understand how collaboration in mental health care organizations takes shape in daily practice.

**Methods:**

We conducted a qualitative study using semi-structured interviews and a focus group, in the setting of the Program for Mentally Vulnerable Persons (PMV). Data were analysed following thematic analysis.

**Results:**

We found three aspect that were considered important in collaboration: commonality, relationships, and psychological ownership. However, our findings indicate a discrepancy between what is considered essential in collaboration and how this materializes in day-to-day practice: collaboration appears to be less manageable than anticipated by interviewees. Our data suggest psychological ownership should be added as value to the interorganizational collaboration theory.

**Conclusion:**

Our study offers a new definition of collaboration and adding “psychological ownership” to the existing literature on collaboration theory. Furthermore, we gained insight into how collaboration between different organizations works in practice. Our research points to a discrepancy between what all the partners find important in collaboration, and what they actually do in practice. Finally, we expressed ways to improve the collaboration, such as choosing between a chain or a network approach and acting on it and re-highlighting the goal of the Program Mentally Vulnerable persons.

**Supplementary Information:**

The online version contains supplementary material available at 10.1186/s13033-023-00584-9.

## Background

Over the last decades, a major change in mental health care has been the transition of patients from an intramural (inpatient) to an extramural (outpatient) setting, via a process called deinstitutionalization. Many countries have responded to this transition with *a system* change from institutional to community-based care [1–3].

The Netherlands also reacted with a *system change*. Since 2015, Dutch municipalities are responsible for support in the field of self-reliance, participation, sheltered housing, and care for mentally vulnerable people [4]. Previously, residents could turn directly to the mental health care institutions, who were reimbursed by the central government. From the point of view of both municipality and psychiatry the key to handling such system changes is collaboration [5, 6].

Collaboration has multiple definitions. One often used is that of Gray and Wood [7]: “*Collaboration occurs when a group of autonomous stakeholders of a problem domain engage in an interactive process, using shared rules, norms and structures, to act or decide on issues related to that domain*”. The field of mental health care distinguishes between two types of collaboration: *interprofessional (IP)* and *interorganizational* (*IO*) [8–14].

IP refers to collaboration *within* a team or organization and focuses on the individual, for whom the following personal characteristics are important: openness, trust, respect, and learning from each other. IP is characterized by a collaborative leadership style, whereby the person in charge works alongside the other employees like a cooperating supervisor.

IO, on the other hand, stands for collaboration *between* organizations, and is often formalized in policy and procedures. This type of collaboration focuses on integral coordination and professional role separation, and demands a greater clarity of roles -- who is responsible for which subject -- than IP. In IO, a sense of identification with the goal of the collaboration is less easily achieved, especially given the differences between organizational cultures and different discourses, as well as issues related to geographical distance and communication. For both interprofessional and interorganizational collaboration, essential aspects of collaboration are communication, trust, respect, power, mutual relationships, shared values and norms, and focus on the vulnerable client.

As several authors have indicated, the fact that deinstitutionalization is centered around persons in need of care, hence vulnerable persons, requires collaboration to be of high quality [15, 16]. This means, for instance, that continuity of care is crucial [15, 16]. Still, organizations such as municipalities, district teams, and the mental health organizations themselves, have not yet broken down the prevailing walls between them, while this is crucial for a successful de-institutionalization, leading to a so-called “wicked problem” [17]. Before deinstitutionalization, parties were highly autonomous, while now they are forced to work together [18, 19].

Being aware of these partitions and taking up its new leading role, the Municipality of Groningen initiated a Program for Mentally Vulnerable persons (PMV) aiming at improving care for these persons by promoting collaboration between the various organizations during the process of transition [20]. Further, the intention of the PMV is to make the switch from system-oriented to client-focused care, meaning the client perspective comes first, a much needed paradigm shift according to Longden [21]. Notably, this is the first municipality-driven project in the Netherlands intended to foster collaborative care for people with a Severe Mental Illnesses (SMI). Yet, how this collaboration will work out in daily practice is still undefined.

From previous research [14], we know that stakeholders find it difficult to move from theory to practice with regard to collaboration. It is pivotal to investigate how parties are currently taking up their roles and how they can improve their collaboration. Thus, in order to improve the situation for mentally vulnerable persons, knowledge is needed of the various responsibilities and roles in the organization and the action perspective of the organizations involved [10]. More specific, which themes about collaboration can be identified in the network of participating organizations. The transition from intra to extramural mental health care has given rise to new dynamics between public and mental health care, introducing a challenge for IP and IO collaboration [13, 22].

Being the first municipality driven project of this kind, makes it interesting to investigate the collaboration between all stakeholders. Results of this research will be instructive for other municipalities, when setting up such a program themselves. This study aims to understand how collaboration between mental health care organizations takes shape in practice and how collaboration in the mental health care system can be improved. We make use of a qualitative combined interview focus group study in which we follow the PMV.

## Methods

### Design

This qualitative combined interview and focus group study, aiming to understand how collaboration between mental health care organizations takes shape in practice, is part of a larger study called Focus, which follows collaboration between different organizations aimed at helping persons with SMI in The Netherlands.

### Setting

In this study, we investigated how collaboration takes shape in practice by following the Program of Mentally Vulnerable persons (PMV). The municipalities in the province of Groningen in The Netherlands, together with mental health care providers, a regional health insurer, and client representatives, have developed this program in 2019, which will run for five years. In addition, three other organizations are involved in the partnership, namely district team, regional public health service and a general practitioner post. The mentally vulnerable persons are people with SMI (i.e., people having a psychotic disorder, a severe mood-disorder, and/or drugs-dependency, have multiple psycho-social problem, and complex needs in the domains of a district team, a mental health institution or other societal organization, such as for housing or financial support).

The main reason to set up the PMV was because the involved organizations noted transition-related problems such as continuity of care, risk of readmission and worsening of symptoms [16]. Another problem was the funding. In the past years, many projects have started, aiming to improve care for people with SMI. These projects each have their own funding source and as a result, the organizations involved became contestants [23]. Lastly, if people with SMI are treated too late, due to the waiting list problem, this leads to more serious illness and higher costs.

The organizations involved in PMV are convinced that working together helps to improve patient care. They agreed to focus on primary and secondary prevention of mental health problems. Further, the intention of the PMV is to make the switch from system-oriented to client-focused care, which stands for listening to the client and act upon it. This switch is necessary according to the organizations involved in the PMV in order to solve the noted problems. The overarching aim of the program is to get to know each other better by working together on projects focusing on improving care for mentally vulnerable persons.

**Fig. 1:**
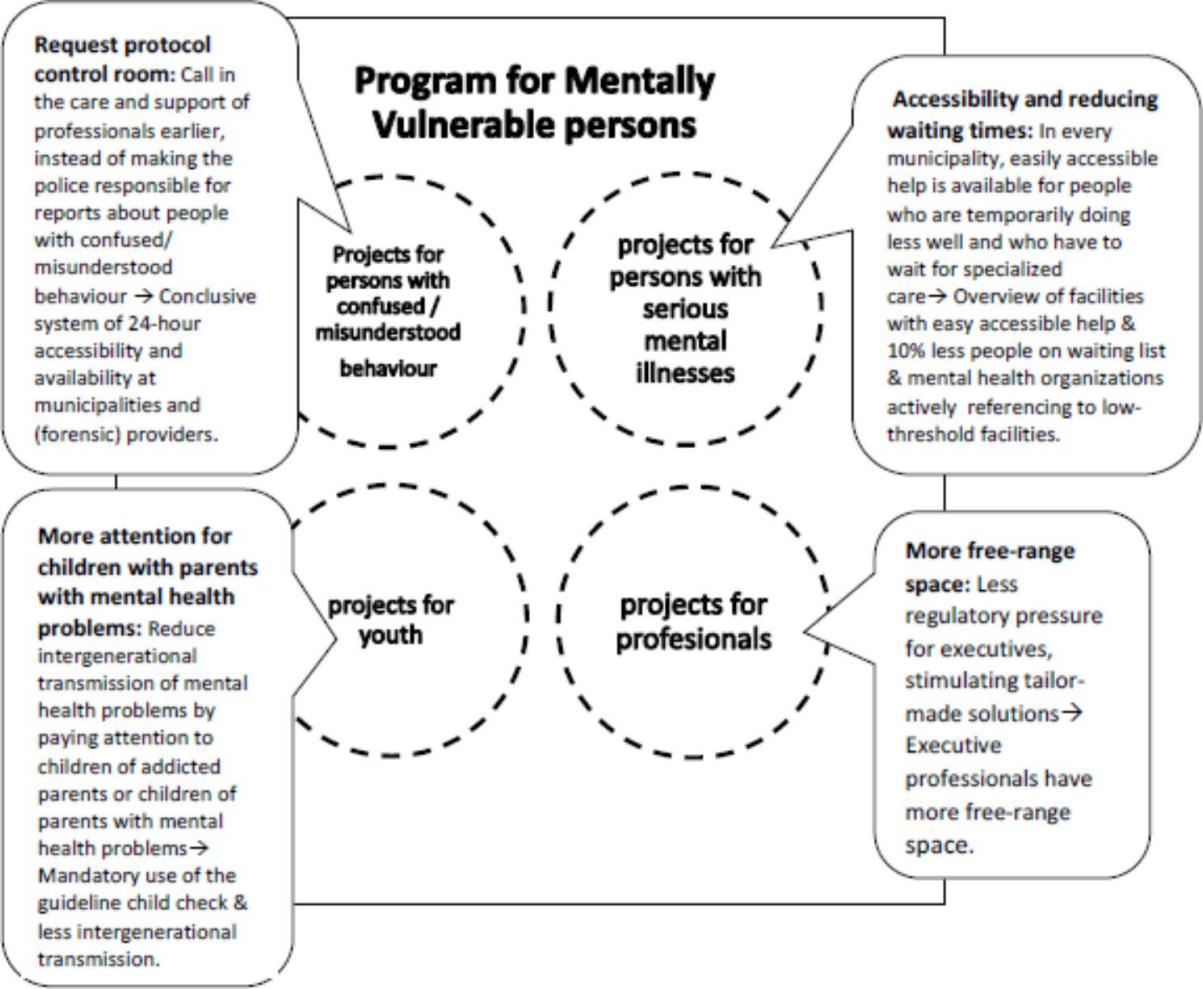
Examples of PMV projects and results

Within the program, a number of projects have been developed that target four different populations: persons with misunderstood behaviour, persons with serious mental illness, youth with mental health problems, and professionals operating in the mental health system. Figure 1 presents some examples of projects.

**Fig. 2:**
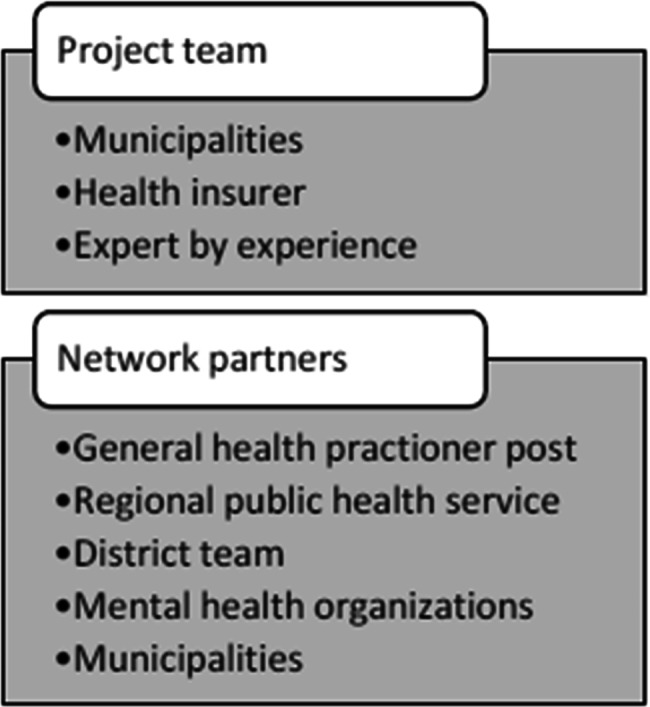
Project team members and network partners

These projects are the responsibility of both the project team members and the network partners. In Fig. 2 information is given about the organizations in the project team and the network.

Project team members regularly discuss the progress of the PMV and inform one another about their own projects that fall under the PMV. The project team members, most of them policy advisors, represent the municipalities, health insurance providers, and clients. One team member is project manager for day-to-day businesses and projects that fall under the PMV.

The municipalities are responsible for the policy and organization of mental health care and they take care of the referrals. The health insurer is responsible for paying the healthcare providers, based on the referral from the municipality.

The network partners, most of them nurses, case managers or psychiatrists, implement the projects of the PMV collaborating with each other to provide care for the target group. The mental health care providers are responsible for providing the right care in the right place. In the collaboration, especially between mental health care providers, all have a different specialty. Thus, one organization focuses on people with an addiction and having psychological problems while others, for example, focus more on people with serious mental illnesses. The district teams provide access to care and provides preventive care. The regional public health service protects, monitors and promotes the health of the inhabitants of the Netherlands. The general practitioner post is present for emergency and medical assistance outside office hours.

### Characteristics of participants

Two types of participants, project members (n = 9) and network partners (n = 10), were included in this study. First, all members of the project team were included, representing municipalities (n = 7), health insurance provider (n = 1), and an expert by experience (n = 1). Next, representatives of all network partners were included (n = 10), representing mental health care providers (n = 5), municipalities (n = 2), district team (n = 1), regional public health service (n = 1) and general practitioner post (n = 1).

We used purposive sampling. The project team manager provided the researcher with the names and contact information of the representatives (n = 19) (see Table 1). All participants received an email with an information letter and an informed consent form from the first author, asking whether they would like to participate in the study. No one refused or dropped out.

**Table 1:** Participant characteristics

Participant nr.	Gender: Female (F) or Male (M)	Project team member	Network partner
1	M	x	-
2	F	x	-
3	M	x	-
4	F	x	-
5	F	x	-
6	F	x	-
7	F	x	-
8	M	x	-
9	M	x	-
2.1	F	-	x
2.2	F	-	x
2.3	M	-	x
2.4	M	-	x
2.5	M	-	x
2.6	M	-	x
2.7	F	-	x
2.8	M	-	x
2.9	M	-	x
2.10	M	-	x

### Data collection

Data were collected in three rounds. First, semi-structured interviews were conducted with members of the project team (n = 7). For these interviews, an interview guide was developed based on literature and researchers’ experiences. The interview guide comprised mainly open-ended questions with subsequent probes to obtain more in-depth information, regarding the following topics: principles and values related to collaboration, expectations regarding development of the collaboration, and experiences with collaboration. If a new topic was raised by the participants, this was added to the interview guide.

Secondly, the results of these interviews were discussed with the project team members in a focus group. The focus group discussion resulted in a description of how they as project team members experienced collaboration, and what they considered important.

Thirdly, representatives of network partners were interviewed (n = 10). Based on the interviews and focus group discussions, the following topics were added to the interview guide: the role of the organization in mental health care, relationships between network partners, their contribution to collaboration, change to client-oriented care, and expectations regarding collaboration. Furthermore, the description of collaboration as formulated in the focus group discussion was verified.

Data were collected and analysed in an iterative process between February and April 2021. This helped us to ask more focused questions in order to elaborate on emerging themes. All interviews, as well as focus group discussions, were conducted online using MS Teams. The duration of the interviews varied from 40 to 50 min; duration of the focus group was 90 min.

SK (MSc, PhD-student, and policy advisor at a municipality, F), was trained in interviewing, and conducted all interviews. At the interviews, only the participant and the interviewer were present. The focus group was moderated by SK; MA (PhD, social scientist, F) and MB (BA, research assistant, F) were present as observers. All interviews were audio-recorded and transcribed verbatim. Along with the interviews and focus group discussion, we also used a reflective logbook for bracketing and writing down notable aspects [24, 25].

As member check, all interviewees were sent an interview summary for approval. The focus group was an interim member check after the first round of interviews. Both after the first round of interviews and after the second round of interviews, results were presented to the project team. They could provide feedback and additions. Data were collected until data saturation was reached, and were then discussed with the research team.

### Data analysis

To analyze the data, we used thematic analysis consisting of six phases [26]. First, after transcription SK read all transcripts and noted initial ideas. Secondly, three researchers (SK, MB, MA) coded data in ATLAS.ti 9. The coding was a iterative process. After a first and second round of reading, involving shading of important passages, we inductively developed the first codes. During the process of coding the transcripts, new codes emerged. Code groups/themes were formed, based on associated codes. The coding tree consisted of sixteen subcodes, thirty-three codes and nine overarching master codes (see supplementary material).

In the next step, SK, MA and MB had consensus meetings in which they searched for themes by interpreting and categorizing the data. This was an iterative process of reading, categorizing and refining. Themes were reviewed, refined and discussed with other members of the research team, LvdK (PhD, researcher and Health psychologist, F) and RB (Professor and psychiatrist, M), until we had reached consensus. In addition, themes were checked with quotations in the data. During the writing process, we defined and named themes. We also related back to the research question and literature to report the data. Finally, in phase six, we looked for illustrative examples of extracts. Illustrative quotations were translated by a native English speaker.

### Reflexivity

The first author (SK) kept a reflexive logbook in which she described her feelings and assumptions regarding the research topic in order to gain insight into these feeling and assumptions and to adjust her actions accordingly [24]. SK assumed at the beginning of the study that the collaboration would have been discussed among the partners of the PMV, as collaboration is the aim of the PMV. Nevertheless, her curiosity about the development of the partnership won out over prejudices and assumptions. SK is a public administration expert and her prior experiences as a policy advisor on protected living for mentally vulnerable persons helped her to understand the mental health system and being able to ask the right questions. As SK is familiar with the research topic and “language”, she has inside knowledge. This could lead to potential biases. SK personally knew, from her role at the municipality, most of the project team members and two of the interviewed network partners, which could have led to prejudices. However, by using bracketing, we have tried to overcome this potential bias [25]. At the beginning of each interview, participants were told why the interview was conducted.

In addition, we interrogated our positionality [27]. We used several methods to ensure our reflectivity. In our research group, we regularly ask each other about how and why we are involved in this research. We discuss this twice a year. Prior to the meetings, we all answer the same questions and thus determine our positionality. These positionalities are discussed during the meetings to learn from each other and to be critical of our actions as researchers.

## Results

Three themes emerged from the thematic analysis: commonality, relationship, and psychological ownership. Below we discuss the values and expectations of the PMV, and its application. The differences in background/profession of the participants, did not appear to influence their experiences.

### Commonality

Commonality is described by participants as striving for shared results, goals, and vision. *“So I’m always looking for that commonality, to cultivate it and then work toward a common goal.”* (R1). Participants regard commonality as important in order to achieve results. In practice, however, commonality turned out to be difficult to manage. Many representatives of the network partners experienced the goals of the PMV as too abstract, and most indicated that not everyone was familiar with the vision and aims of the program. *“Then it (the goal) has to be more concrete and now it’s often still so highly abstract that people may also be reluctant to cooperate ….”* (R2.3) Furthermore, the project team had the impression that the network partners had varying interests, an impression shared by some representatives of the network partners themselves. Finally, an interviewee indicated that much more progress is needed when it comes to joint responsibility: “*That’s where we still have a long way to go, I notice, yes, before we feel a joint responsibility for things.*”(R 4).

### Relationships

The second theme, relationships, consists of three subthemes: (a) the relationships between the network partners and project team members themselves, (b) the position of the client within the PMV and (c) personal qualities.

All participants recognized the importance of their mutual *relationships*. The network partners all contributed to the mental health care network. Each expected the others to look beyond their own organizations and roles. R2.10 states: “*Well I think it’s important that it’s very clear who does what and who does…, which isn’t done by a particular chain partner. So I think that’s very clear, that you’re explicit in that*.“. For example, the members of the project team expected the network partners to take a leading role; however, in practice this was not the case. The project team members also reported that some network partners did not commit themselves to the results of the PMV. The metaphors used to describe collaboration were also diverse: e.g., island structure, or a monopoly game in which everyone had different interests. Conversely, when interviewed, network partners indicated that they had expected more information and advice from the members of the project team. The network partners experienced their mutual relationships as unsustainable and varying in quality.

All interviewees, both network partners and project team members, believed that *clients* should play an important role, and in fact should be at the center of the collaboration within the PMV. In practice, however, according to all partners the clients appeared to play little or no role in the collaboration, or, if they were in the picture, they played a subordinate role: *“Ideally, he (the client) is central, …., but when you look at the picture, I sometimes have the feeling that you’ve already gone past the client, that he is behind you.”(R 5).* According to another interviewee, whether you operated at executive or management level affected how you looked at the client: *“We start with a plan that’s organized with the client and not above the client. So, it is very much…, we very much follow the movement of clients themselves. So, at the executive level we are really next to them. Beyond that, it becomes very difficult, because then you see that not many clients are represented.“(R 8).* This was confirmed by a large proportion of the managers, who indicated that they seldom came into contact with clients.

Participants mentioned a number of *personal qualities* that were important to build strong relationships, especially while collaborating, such as learning from each other, and being curious. All interviewees also emphasized the importance of having confidence in each other and trusting each other. However, in practice trust was experienced in varying degrees: “*Yes, trust in each other, yes, certainly that is a key concept. You have to trust each other to work well together. If you do not have trust you can pretend to work together or you can act together, but then you always have conflicting interests*.”(R 1.). Transparency was another quality that interviewees considered relevant for collaboration, as well as dedication and curiosity. Further, learning from each other did not seem to go very well in practice. R 2.8 said: “*And let’s just all look together, out of curiosity, at each other’s group, what can we do about this for each other?“.*

### Psychological ownership

The theme *psychological ownership* emerged inductively from the interviews with the network partners. The project team was therefore not questioned about this. Almost all network partners considered psychological ownership important. According to interviewees, psychological ownership is a basic condition for being involved and wanting to contribute to the collaboration. Moreover, a sense of urgency seemed to promote psychological ownership. It made collaboration easier and smoother: *“If you feel ownership … then people are more easily or more quickly inclined to say yes.”(R 2.3)*.

Most network partners indicated that they did not feel psychological ownership of the PMV as a whole: *“We play a role, but I don’t feel like we have real ownership. No. It’s not alive.“(R 2.9).* However, some indicated that they did feel psychological ownership in specific projects in which they or their organization were involved: *“Well, in part. I do feel ownership when it comes to the bedding of the expertise (*of the organization involved) *and positioning it well.“(R 2.5).*

The lack of psychological ownership and unfamiliarity with the PMV are illustrated by the following anecdote from the logbook of SK. One participant responded to the invitation to participate in the interview as follows: “*The PMV? What is that?“.* However, when the PMV project in which the organization was participating was explained, he understood what it meant.

### Definition collaboration

At the start of this research, we discovered that the project team had not specified how collaboration could be carried out. We therefore decided to ask them to explain their values and expectations regarding collaboration. A definition was first formulated, based on the interviews with the project team, and then refined in the focus group. This definition was subsequently checked in the interviews with the network partners. Based on these findings, we arrived at the following definition: “*Collaboration is about the pursuit of common goals and results and a shared vision, with curiosity, trust in each other, dedication, learning from each other and transparency in everyone’s interests, all of this in close connection with the network partners and clients.”*.

## Discussion

In this study we examined how collaboration between organizations changed in day-to-day life during the implementation of a new program to improve care for mentally vulnerable people. The various involved organizations involved reported three themes of collaboration: *(i)* *commonality*, *(ii)* *relationships*, and *(iii) psychological ownership*. This last aspect, with regard to collaboration theory, is new. Furthermore, we observed a disconnection between what is considered crucial in collaboration and what is actually carried out in practice.

### Collaboration themes

The first two themes – *commonality* and *relationships* - can also be found in previous studies on collaboration. *Commonality*, striving for shared results, goals and vision, is one of the facilitators of collaboration [5, 28, 29]. In our case, however, at the start of the program the project team had not yet discussed what the collaboration should deliver, and therefore, a sense of commonality was less present.

Our study indicated that *relationships between all partners* are about knowing who has which role, thereby creating clear expectations about what each has to offer. In the literature, this way of perceiving relationships is described as role definition, or transparency about where everyone’s role begins and ends [30].

In the interviews, the position of the patient or client within the system, was often referred to as centre of the system. In literature this central position is described as person-centred [31–33].

*Personal qualities* like curiosity, dedication, learning from each other, and being transparent about interests are important personal elements of collaboration [8, 11, 12, 28]. A lack of trust and confidence in each other forms a significant barrier to collaboration [5]. In fact, lack of trust can get organizations to sabotage the project outcome, according to game theory principles [34]. When it comes to game theory, team members will always choose their collaborative strategy based on the size of their investment related to the possible gain. If they believe they can trust other team members, they will commit to the mutual goal and strive towards collaboration. However, if trust is lacking, they are far less likely to invest in collaboration because they cannot rely on the other team members’ investments and therefore are not sure of the outcome [35].

*Psychological ownership* emerged inductively from the data as a new theme, mentioned only by representatives of the network partners. In organizational literature, ownership emphasizes the ownership of goals, and refers to shared responsibility in the process of reaching these goals [13, 28, 36]. In the present study, ownership is more in line with the concept of psychological ownership, a state of mind in which the individual feels that the target of psychological ownership belongs to him or her (what do I feel is mine). Psychological ownership is about feeling a deeply grounded connection with the subject of collaboration, in this study, the PMV itself and the projects of the PMV. It differs from commitment (shall I remain a member), identification (who am I), or internalization (what do I believe) [37]. It is facilitated by communication and by giving time and attention to the goal [38].

Our definition of collaboration, “*Collaboration is about the pursuit of common goals and results and a shared vision, with curiosity, trust in each other, dedication, learning from each other and transparency in everyone’s interests, all of this in close connection with the network partners and clients.”*, emphasizes the connection between all of those engaged in the collaboration, and, in contrast to the definition of Gray and Wood, is not problem orientated [7]. Furthermore, our definition points out the importance of personal qualities in collaboration. Finally, it emphasizes an active involvement of network partners and clients.

### Possible explanations disconnection

It was not striking to find a discrepancy between what people find important in collaboration and network development and how they act in day-to-day practice [14, 35]. Clearly, everyone wants to commit to collaboration, but practice shows that collaboration does not happen by itself. We are interested in the *why* of this disconnection. To accentuate this disconnection, the metaphors for collaboration collected during the interviews are revealing. We heard of “the monopoly game” which stands for all partners having different interests, and “the island structure”, indicating that organizations are not interconnected. We also were interested in the position reserved for clients in the metaphors. All answers indicate that the client is insufficiently visible. These metaphors illustrate that collaboration faces serious issues. We present four explanations for this disconnection: differences between interprofessional and interorganizational collaboration, lack of psychological ownership, innovation ecosystem theory and several discourses.

First, our study shows clearly how project team members and network partners use *different collaboration styles.* Collaboration in the project team follows the line of interprofessional collaboration, whereas collaboration between network partners follows the line of interorganizational collaboration. These styles differ when it comes to types of leadership and the handling of rules and policies. The use of different ‘language’ to describe collaboration makes miscommunication inevitable.

The second explanation can be the *lack of ownership*. Our study makes clear that psychological ownership is a relevant condition for collaboration. Psychological ownership occurs when an individual can exercise control, and has an intimate relationship with the goal [39]. These are also features of interprofessional collaboration. Our results indicate a lack of psychological ownership, the absence of a clear goal, and the feeling of having no influence or control. Our study shows that almost all interviewees consider psychological ownership significant. This feature should therefore be incorporated in interorganizational collaboration theory to complement the concept. Furthermore, until now psychological ownership as a concept has been applied only to interprofessional collaboration, and not to interorganizational collaboration [38].

Looking through the lens of the innovation *ecosystem theory*, we can provide a third explanation for the disconnection between theory and practice with regard to collaboration [40]. The starting point of the ecosystem theory is that the coherence of the system is central, in which all elements influence each other [41]. Distinctive aspects of the ecosystem theory could offer an explanation for the disconnection that we found, these are ‘context’; ‘ecosystem goal’; ‘stakeholders’ and ‘organizational structure’ [42, 43].

Deinstitutionalization has altered the *context* of the ecosystem. Whereas in the past mental health care was solely responsible for health care access, now the municipality is the most important initiator and regulator. This creates considerable challenges and problems, due to the transition from intra- to extramural care, which has not been properly managed [21]. Critical issues like bridging the gap between mental and public health; changing goals, roles, and relationships; and the medical predominance model, have not yet been resolved.

In our study, the *ecosystem goal* focuses on a conversion from system- to client-orientated care. Our study shows that this goal was not clearly stated for the network partners. The absence of a clear goal hampers the genesis of psychological ownership. If partners do not know why they should collaborate, it is difficult for them to feel the collaboration as their own. Consequently, they are less inclined to commit themselves to the PMV, with the result that goals are less well achieved.

The *stakeholders* in this study were the project team and the network partners. To our surprise, the client, the most important stakeholder in the ecosystem, the one for whom the entire collaboration was established, was not in sight. In the interviews the client was mentioned but was not the first topic to come up. We can thus conclude that the switch from system-oriented to client-focused care is not yet complete.

**Fig. 3:**
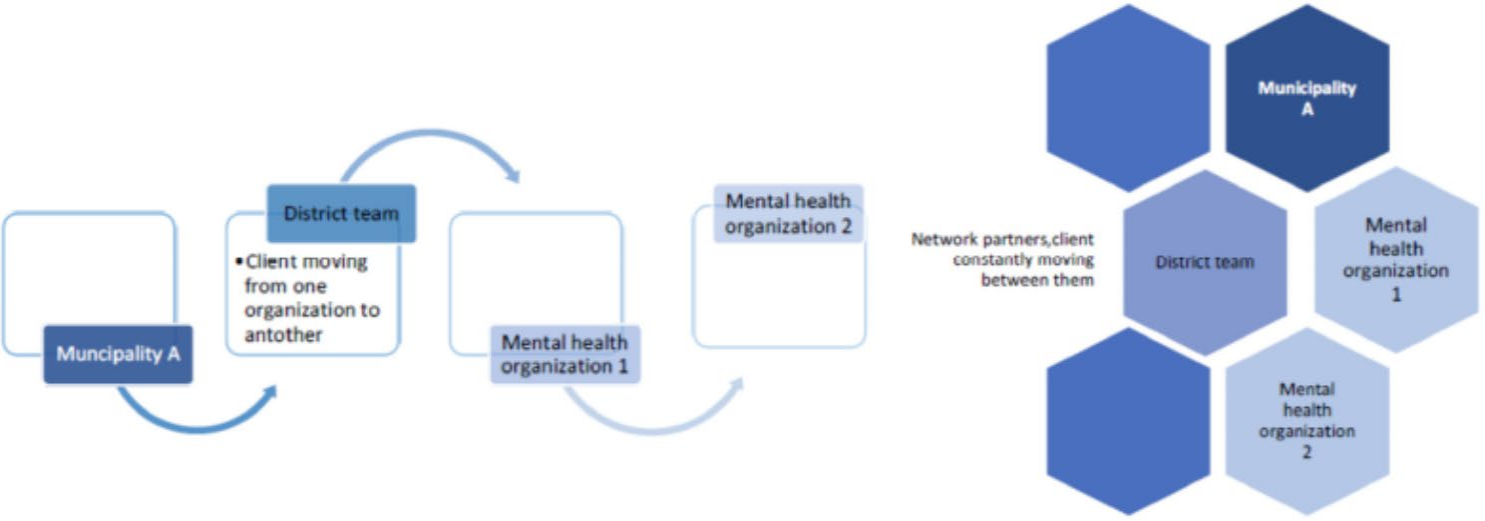
Chain and network partners

Organizational *structure* in mental health care is gradually moving from a chain structure to a network structure. The terms “chain partners” or “network partners” have different connotations. The word “network” evokes another feeling than the word “chain,” and the two structures encourage different types of behavior. While working as chain partners, clients may disappear from sight, and responsibility be handed over to another professional/organization. A network structure requires continuous alert behavior, keeping an eye on the client, as the responsibility for the client is constantly shifting (see Fig. 3). Knowing that the client is more or less out of sight, and also knowing that this is more likely to happen between chain partners than between network partners, we can conclude that the ambition of the PMV to be a network structure has thus far been thwarted.

Finally, in mental health care organizations several discourses are used, such as the biomedical discourse, the institutional discourse, or the multidisciplinary discourse [44]. These varying approaches can make collaboration difficult. To cope with this, each network partner must take stock of the other; each must invest in the relationship. A lack of such investment may explain the unstable collaboration [5]. Change in an uncertain situation can eventually bring about an evolution. Such an evolution necessitates another discourse -- responsibilities, positions and roles will change, both for mental health care providers and municipalities. Interestingly, Ten Dam and Waardenburg explored how professionals use respective logics to give meaning to changing elements in their working environment [45]. They found professionals to be quite adaptive in the use of logics, as they seem to able to choose the best logic for each specific situation.

### Implications

Continuing our consideration of how to improve collaboration in the PMV, we can formulate several implications for *practice*. First, based on comparison with the innovation ecosystem, implementation of the PMV calls for attention to various points: for example, being aware of the context of the ecosystem, the client -where is he and where do you want him to stand-; choosing between a chain or network approach and acting on it; being conscious of the most used discourse and logic; and re-emphasizing its goal. Interventions like professional discussion could be used to develop qualities considered important for strong collaborative relationships and to consider the above recommendations. Secondly, it is important to pay attention to the differences and similarities interprofessional and interorganizational collaboration. Thirdly, it is valuable to work on psychological ownership because this is lacking. Psychological ownership starts with a practicable goal. Finally, it is advisable to perform a network analysis, a method that investigates the relations between all partners in the network and combining it with a game theoretic approach [34]. Network analysis makes clear where elements are missing, like linking pins or connections between organizations, and indicates the ambiguities in the relationships between network partners. For example, without a linking pin - someone who understands how the various organizations work - it is difficult to transfer information [46]. Game theory is supplementary to network analyses because even if everyone indicates that they want to work together in the network, the game theory conditions are not always met to achieve collaboration. Network analyses reveals where possible imbalances occur. Secondly, the costs can be greater than the profit, therefore it is necessary to have insight into the network and understand how the cost-benefit analysis turns out [35].

In the context of our research, we returned our results to the interviewees. Sharing our findings gives our research subjects the opportunity to adjust their working methods. We are curious to see the possible results of a repeat of this research in two years’ time.

For further *research*, we suggest building a collaboration model, using the relevant themes. It could be interesting to study the correlation between different themes, to gain insight into how to improve collaboration. We argue that mutual relationships and personal qualities like trust and curiosity can help to enhance commonality. Learning from each other, being curious, having confidence in each other – these things can create a feeling of mutual connection. Our results also suggest that psychological ownership should be added as a basic condition when studying collaboration. A new model based on these assumptions can complement existing models. Further, we recommend a similar study of another collaboration to strengthen and evaluate our results. Finally, the ecosystem theory proves to be of value, as it covers all important aspects. It may therefore be relevant to use this theory more often.

### Strengths and limitations

This research followed the PMV for four years, from its very beginning. This study can be seen as a baseline measurement. The research studied the values and expectations related to collaboration, and how this work out in practice. To the best of our knowledge, this was the first time for such a diverse network of mental health care providers, district teams, municipalities, and a provider of health insurance, to be a subject of research. Further, including only people with a positive attitude towards the PMV and its collaboration could have biased our study. However, based on the results, this does not seem to be the case. Finally, as we described the PMV in a specific local situation, the results may not be generalizable to other regions. Nevertheless, as this PMV is the first in the Netherlands, our research can be a guideline to help other municipalities to avoid pitfalls.

## Conclusion

Exploring collaboration, we found a significant role for *commonality*; r*elationships;* and *psychological ownership.* Our study offers a new definition of collaboration, adding “psychological ownership” to the existing literature on collaboration theory. Furthermore, we aimed to gain insight into how collaboration between different organizations works in practice. Our research points to a discrepancy between what all the partners find important in collaboration, and what they actually do in practice. The ecosystem theory, together with the interprofessional and interorganizational collaboration theory, and the concepts of psychological ownership, discourses, and logics, proved useful in explaining this disconnection. The ecosystem also helped to elucidate the functioning of a complex system like the PMV. Finally, we expressed ways to improve the collaboration, such as choosing between a chain or a network approach and acting on it and re-highlighting the goal of the PMV.

## Electronic supplementary material

Below is the link to the electronic supplementary material.

Additional file: Table S1. Code tree

## Data Availability

The datasets generated and/or analysed during the current study are not publicly available due privacy of the participants, but are available from the corresponding author on reasonable request.

## List of abbreviations

IP: Interprofessional
IO: Interorganizational
PMV: Program of mentally vulnerable persons
SMI: Severe mental illnesses
F: Female
M: Male
SK: Suzanne Kroon
MB: Meike Bak
MA: Manna Alma
JvdK: Lian van der Krieke
RB: Richard Bruggeman
