# Supplementary material for: Values and practice of collaboration in a mental health care system in the Netherlands: a qualitative study
Source: Int J Ment Health Syst. 2023 Jun 8;17:15. doi: 10.1186/s13033-023-00584-9 (PMC10249202; doi:10.1186/s13033-023-00584-9)
Supplement: Supplementary file 1 — Additional file: Table S1. Code tree [file 13033_2023_584_MOESM1_ESM.docx]

| **Supplementary material Ref: Submission ID b1239dc4-b369-4d25-838c-87dbe8b7e39a : code tree** | | |
| --- | --- | --- |
| Code group | Codes | Subcodes |
| All about chain partners | Awareness PMV chain partners  Contribution to PMV collaboration  Ownership Motivation  Involvement/cohesion chain partners PMV  Conversion system-client  Respondent role/function | - |
| Collaboration between chain partners | Collaboration between municipality-chain partners  Collaboration between mental health care providers - chain partners  Collaboration between national partners  Collaboration between police and judicial chain partners  Collaboration between housing association-chain partners  Collaboration between health insurer-chain partners | - |
| Important in collaboration | Important in collaboration common  Important in collaboration-dealing with each other  Important in collaboration-other  Important in collaboration-trust | - |
| Current State of collaboration 2021 | Current state of collaboration 2021 features  Current State of Collaboration 2021-Program Manager | - |
| Motivation | Motivation-career path  Motivation-function-related  Motivation-personal motive  Motivation knowledge and skills | - |
| Problems & Challenges 2021 | Problems & Challenges 2021 Issues  Problems & Challenges 2021 Challenges | - |
| Roles | Role client/resident | Role client/resident interest  Role of client/resident-where is he/she  Role client/resident - what action needed |
|  | Role national | Role of national partners  Role of national laws and regulations |
|  | Role research | Role research |
|  | Role partners through project team | Role partners through project team-enthusiasm  Role of partners through project team-municipality  Role partners through project team-relationships |
|  | Role project team member | Role project team member-implementation  Role project team member-create conditions  Role project team member-experience |
|  | Task/role Program manager | Task/role Program manager-addressing role views  Task/role Programme Manager management  Task/role Connecting Program Manager parties  Task/role Programme manager citizen spokesperson |
| Expectations & hope | Expectations & hope-overall  Expectations & hope-what is needed | - |
| Miscellaneous | Aim PMV  Check collaboration definition  Need overall  Ideas about care  Metaphor/image collaboration | - |
